# Sexual asthenia: Tradamixina versus Tadalafil 5 mg daily

**DOI:** 10.1186/1471-2482-12-S1-S23

**Published:** 2012-11-15

**Authors:** Fabrizio Iacono, Domenico Prezioso, Ester Illiano, Giuseppe Romeo, Antonio Ruffo, Bruno Amato

**Affiliations:** 1 Department of Urology – University Federico II of Naples, Via S. Pansini, 5 – 80131 Naples – ITALY; 2 Department of General, Geriatric, Oncologic Surgery and Advanced Technologies, University “Federico II” of Naples, Via Pansini, 5 - 80131 – Naples, Italy

**Keywords:** Testosterone deficiency, libido, erectile dysfunction

## Abstract

**Background:**

Reduced libido is widely considered the most prominent symptomatic reflection of low testosterone (T) levels in men. Testosterone deficiency (TD) afflicts approximately 30% of men aged 40-79 years. This study seeks to evaluate the effect of a new natural compound “tradamixina “in order to improve male sexual function in elderly men, particularly libido and possible erectile dysfunction, versus administration of tadalafil 5 mg daily.

**Methods:**

Seventy patients (67.3± 3.7 years) with stable marital relations and affected by reduced libido, with or without erectile dysfunction were recruited. They were randomly separated in 2 groups A-B of 35. Group A was administered twice a day a new compound “Tradamixina” (150 mg of Alga Ecklonia Bicyclis, 396 mg of Tribulus Terrestris and 144 mg of D-Glucosamine and N-Acetyl-D-Glucosamine) for two months, while Group B was administered tadalafil 5 mg daily, for two months. At visit and after 60 days of treatment patients were evaluated by means of detailed medical and sexual history, clinical examination, laboratory investigations (Total and Free T), instrumental examination (NPTR- nocturnal penile tumescence and rigidity test- with Rigiscan). Patients completed a self-administered IIEF questionnaire (The international index of erectile function) and SQoLM questionnaire (Sexual quality of life Questionnarie-Male). The results pre and post treatment were compared by Student t test (p<0.005).

**Results:**

After 2 months of treatment in group A serum TT levels (230±18 ng/dl vs 671±14 ng/dl ) and FT levels(56± 2.4 pg/ml vs 120± 3.9pg/ml) increased, while in group B serum TT levels (245±12 ng/dl vs 247±15 ng/dl ) and FT levels(53± 0.3 pg/ml vs 55± 0.5pg/ml) increased not statistically significant. The patient’s numbers with negative NPTR improved after treatment in group A and B (15 vs 18 and 13 vs 25 respectively). The IIEF total score in group A increased after treatment with tradamixina (15±1.5 vs 29.77±1.2); the IIEF total score in group B increased slightly (12±1.3 vs 23.40±1.2). The SQoLM total score improved in both groups (A:16±2,3 vs 33±4,1 and B: 16±3,4 vs 31±2,1).

**Conclusion:**

The treatment twice a day with “Tradamixina” for 2 months improved libido in elderly men without side effects of Tadalafil.

## Background

Reduced libido is widely considered the most prominent symptomatic reflection of low testosterone (T) levels in men [1,2] . Testosterone deficiency (TD) afflicts approximately 30% of men aged 40-79 years, with an increase in prevalence strongly associated with aging and common medical conditions including obesity, diabetes, and hypertension [3]. Although decreased libido is a concern often expressed by aging patients [4], it is difficult to measure comprehensively, being multifactorially determined and associated with both psychosocial and organic factors [5]. This phenomenon of hypogonadism due to aging has also been described as testosterone deficiency syndrome, late-onset hypogonadism, and andropause. Symptoms of this condition resemble those of ‘normal’ aging and include changing body composition (osteopenia, increased adiposity, decreased muscle mass), decline in energy and stamina, decreased cognitive function, decreased libido, and erectile dysfunction [6], systolic hypertension, carotid artery-wall thickness, increased abdominal visceral-fat mass, insulin resistance, reduced HDL concentrations, postprandial somnolence, impaired quality of life and depressive mood [7]. Particularly in elderly men, decreased libido and impotence present a common and important clinical problem. Sexual dysfunction, encompassing erectile dysfunction, ejaculatory disorders and loss of libido, is highly prevalent in ageing men and can have substantial adverse effects on their quality of life [8-11]. Nevertheless several studies have shown that there is no clear association between testosterone levels and erectile function [9-11]. In fact although androgen replacement has been shown to enhance sexual function in many elderly men with low testosterone levels [12,13] it cannot be assumed that androgen deficiency is responsible for impaired potency in older men when testosterone levels are in the normal range. Furthermore, no data is available to judge the effect of falling testosterone levels as men age when testosterone concentrations are still within the normal range [14]. After age 50 the percentage of men who had experienced their first problems with erection increased sharply—26% in men age 50 to 59 years and 40% in men age 60 to 69 years [16-18]. The prevalence of erectile dysfunction increased with increasing age, infact androgens are essential for the development, growth and maturation of the erectile tissues. In the animal model testosterone suppression led to corpora cavernosum atrophy with concomitant structural alterations of the dorsal nerve of the penis, endothelial alterations, reduction of the smooth muscle component and increase in the deposition of extracellular matrix and cavernosal fibrosis [19,20]. Unlike the libido, men with a healthy lifestyle and no chronic disease had the lowest risk for erectile dysfunction; the greatest difference was seen for men 65 to 79 years of age. The absolute risk for erectile dysfunction was approximately 10% higher at all ages for men with comorbid conditions compared with healthy men [21,22]. Testosterone levels in men begin to decline in the late third or early fourth decade and diminish at a constant rate thereafter [23]. There is no universal agreement regarding the exact definition of hypogonadism. However, it is generally accepted that hypogonadism refers to the presence of persistently low circulating testosterone compared with the normal range derived from healthy young and middle-aged men. This range is approximately 300-1000 ng/dL or 10.4-34.7 nmol/L in most assays of serum total testosterone [24], although wide variation may exist between different commercial assays [24,25]. In the elderly, the diagnosis of hypogonadism is sometimes problematic because of the difficulty to know to what extent the previous features are due to aging, hypogonadism, or both. Longitudinal studies, such as the Massachusetts Male Aging Study, suggest that total testosterone decreases at a rate of about 1.6% annually, with a concomitant 1.3% annual increase in SHBG after age 40 [23]. The fraction of circulating testosterone that is bound to SHBG is inactive, so its increase results in even lower levels of bioavailable testosterone. An estimated 30% of men aged 70-79 have low serum total testosterone and approximately 70% have low bioavailable testosterone levels [27]. The mechanisms causing lower testosterone levels in healthy elderly men include a decreased responsiveness of Leydig cells to luteinizing hormone (LH) and a blunted compensatory elevation of LH to the lower testosterone levels [28]. This study seeks to evaluate the effect of a new natural compound “tradamixina “ made of Alga Ecklonia Bicyclis, Tribulus Terrestris and D-Glucosamine in order to improve male sexual function in elderly men, particularly libido and possible erectile dysfunction ,versus the use of tadalafil 5 mg daily. Seven phlorotannins were isolated and characterized from an edible marine brown alga *Ecklonia Bicyclis*, along with three common sterol derivatives (fucosterol, ergosterol, and cholesterol) according to the comprehensive spectral analysis of MS and NMR data. Compounds 7-phloro eckoland and ,6,6′-bieckoll in phlorotannin derivatives were obtained for the first time with their high respective yields. No bioactive reports of the compound Fucodiphloroethol G were found to date [29]. Ecklonia bicyclis has radical scavenger activity 10-100 times more powerful than any other polifenol terrestris plants, which have only 3-4 fenolic and rings that are commonly considered among the most effective antioxidant molecules. Common polyphenols are soluble in water also and have a relatively short half-life introduced into the body. All phlorotannins had antioxidant properties in vitro, especially, compounds 6,6′-bieckol which showed the significant activities compared to the other phlorotannins [29]. This capacity is useful like anti-aging and antioxidant, in the aging sexual process. The protodioscin is a steroidal saponin, which is about 45% of the extract obtained from aerial parts of Tribulus Terrestris. Thanks to its particular steroidal structure it has an androgen-mimetic action, binding and activating the receptor of testosterone. So this substance is able to increase the endogenous production of testosterone, dihydrotestosterone, a hormone luteinizing hormone (LH), dehydroepiandrosterone (DHEA) and dehydroepiandrosterone sulfate (DHEA-S). Because of these effects in experimental animals there is an increase in spermatogenesis and the frequency of matches. In the rabbit in particular it has been shown that the compound stimulates the release of nitric oxide (NO) by vascular endothelium of the corpora cavernosa thereby having a pro-erectile effect. The mechanism behind this effect appears to involve the pathway of steroid hormones. Although in humans protodioscin is used for the treatment of erectile dysfunction [30,31]. In a placebo-controlled study on a group of young volunteers, were detected serum levels of testosterone, androstenedione and luteinizing hormone were detected after administration of Tribulus terrestris at doses of 10 and 20 mg / kg. After 4 weeks of treatment, these values were similar to those of untreated [33]. Biovis contains polymers of d-glucosamine and n-acetyl-d-glucosamine that act both on non-adrenergic and non-colinergic system (NANC) and on endothelial cell system as a strong nitric oxide synthetase (NOS) stimulator [34]. The phosphodiesterase type 5 (PDE5) inhibitor tadalafil can be administered on demand (5−20 mg) or once daily (2.5-5 mg) to treat erectile dysfunction (ED). Once-daily treatment largely obviates the need to plan sexual activity within a narrow therapeutic window after dosing. A number of recent studies, utilizing the International Index of Erectile Function−Erectile Function (IIEF-EF) domain [35], as well as the Sexual Encounter Profile (SEP) questionnaire, as outcomes, have supported the efficacy of once-daily tadalafil [36-40].

## Methods

A double-blind, randomized design trial was conducted from January 2012 to May 2012, at our Department of Urology. Inclusion criteria were: over sixty years of age, male patients with stable marital relations and affected by reduced libido (RL), with or without erectile dysfunction (ED). Exclusion criteria were: diabetes mellitus and other metabolic disorders (impaired glucose tolerance, impaired fasting glucose, metabolic syndrome and congenital or acquired dyslipidemia), obesity, alcoholism, smoking, hypertension, cardiovascular disease, Neurogenic syndrome (multiple sclerosis, multiple atrophy, Parkinson’s disease, tumors, stroke, disk disease, spinal cord disorders, Polyneuropathy, uraemia), Peyronie’s disease, penile fracture, congenital curvature of penis, micropenis, hypospadias, epispadias, hyperprolattinemia, hyper and hypothyroidism, Cushing’s disease, secondary hypogonadism, or hypogonadotropic hypogonadism, drug assumption (antihypertensives,, nitrates, antidepressants, antipsychotics, antiandrogens, antihistamines, heroin, cocaine and methadone) radiotherapy (pelvis or retroperitoneum) and lower pelvic surgery (oncological pelvic surgery, lower urinary and genital tract surgery) and depression.. We enrolled 70 patients, mean age was 67,3± 3,7 years. They were randomly separated in 2 groups A-B of 35 patients. Group A was administered twice a day a new compound “Tradamixina” (150 mg of Alga Ecklonia Bicyclis, 396 mg of Tribulus Terrestris and 144 mg of D-Glucosamine and N-Acetyl-D-Glucosamine) for two months, while Group B was administered tadalafil 5 mg daily, for two months. At visit patients were evaluated by means of detailed medical and sexual history, clinical examination, laboratory investigations (Total and Free Testosterone), and instrumental examination (NPTR- nocturnal penile tumescence and rigidity test- with Rigiscan). Patients completed a self-administered IIEF questionnaire (The international index of erectile function) and SQoLM questionnaire (Sexual quality of life Questionnaire-Male). The therapeutic effects were evaluated by IIEF, SQolM, Rigiscan and total ad free testosterone levels after 60 days of treatment. Written informed consent was obtained from all patients. We asked all patients to complete the International Index of Erectile Function (IIEF) questionnaire: the IIEF domain was calculated and ED grading was determined: absent of ED (EF score 26 to 30), mild ED (EF score 17 to 25), moderate ED (EF score 11 to16) and severe ED (EF score < 10) [40]. We asked all patients to complete the Sexual quality of life Questionnarie-Male (SQoLM) (Pfizer Ltd, Sandwich, UK UK English): this questionnaire consists of a set of statements, each asking about thoughts and feelings that the man may have about his sexual life. The statement may be positive or negative. They were asked to rate each statement according to how much they agreed or disagreed. The Sexual Quality of Life questionnaire Male (SQoL-M) contains 11 items each with a 6-point Likert-like response scale ranging from *‘completely agree’* to *‘completely disagree’*. Items are scored 1-6 (worst to best) and will be scored from *Completely Agree* = 1 to *Completely Disagree* = 6. To allow easy comparisons with other measures, raw scores will be transformed onto a standardised scale of 0 to 100 using the following formula. *Scale Score* = *the sum of the component items* (*minus*) the lowest possible score* 100/ possible raw score range. Higher scores imply greater quality of life. For those questionnaires with missing values, a total score will be calculated for the individual if at least 50% of the items have been completed (at least 6 items) using the equation above. Any questionnaires with >50% missing items will be removed from the analyses. All patients underwent nocturnal penile tumescence and rigidity test (NPTR) with Rigiscan for three consecutive nights. Normal erectile function was defined with the recording of at least one erection (70 out of 100% tip rigidity lasting for at least 10 min during either night) [41]: we considered as Negative Rigiscan (NR) in patients who had an erectile event at least 70% rigidity recorded on the tip of the penis, which lasted for 10 minutes or more, and Positive Rigiscan (PR) in the remaining patients. We practiced this test with the intent of differentiating organic and psychogenic erectile dysfunction [42]. Serum testosterone concentrations were measured using the DPC Coat-A-Count RIA kit, which has an intra- and interassay coefficient of variation (CV) of <10% while sex hormone-binding globulin (SHBG) were measured by ELISA technique (DRG Diagnostics, Marburg, Germany). We calculated the measurement of free testosterone from measured total testosterone and SHBG. These measurements were obtained between 07.00 am and 11.00 am. There is general agreement that total testosterone levels above 12 nmol/L (346 ng/dL) or free testosterone levels above 250 pmol/L (72 pg/mL) do not require testosterone substitution. Similarly, testosterone supplementation should be started according to the reference levels given in the recommendations of the ISA-ISSAM-EAU [10] when serum total testosterone levels are below 8 nmol/L (231 ng/dL) or free testosterone levels are below 180 pmol/L (52 pg/mL) and when serum total testosterone levels are between 12 and 8 nmol/L or free testosterone levels are between 250 and 180 pmol/L in patients with symptoms of testosterone deficiency. So we considered Normal Testosterone (NT) patients who had total testosterone levels above 12 nmol/L (346 ng/dL) and free testosterone levels above 250 pmol/L (72 pg/mL), and Low Testosterone (LT) patients who had testosterone levels below 12 nmol/L (346 ng/dL) and free testosterone levels below 250 pmol/L (72 pg/mL). The study population was divided into two groups: patients treated with tradamixina and patients treated with tadalafil, and compared for total score IIEF pre and post treatment , SQolM results pre and post treatment, mean total and free testosterone levels pre and post treatment, Rigiscan results pre and post treatment by Student t test (p<0.005).

## Results and discussion

**Fig1:**
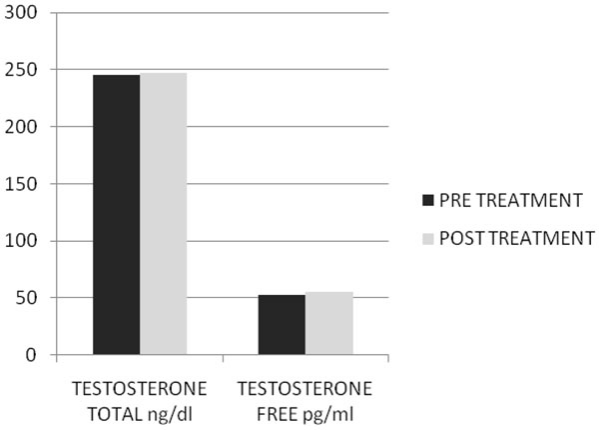
Serum Total Testosterone Level Pre and Post Treatment with Tadalafil 5mg daily.

**Fig.2:**
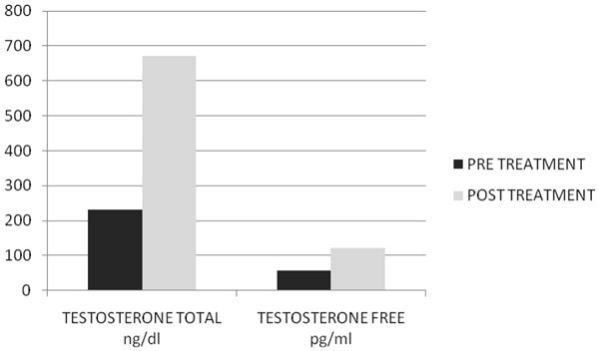
Serum Total Testosterone Level Pre and Post Treatment with Tradamixina.

**Fig. 3:**
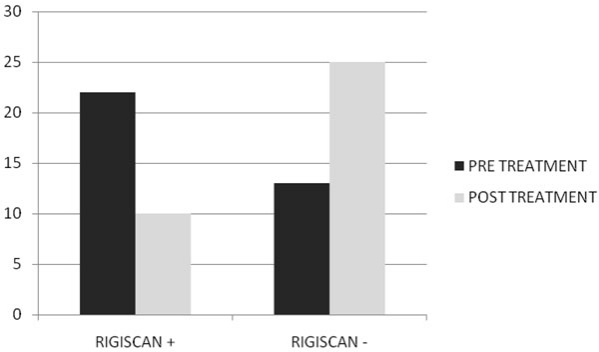
Rigiscan Results Pre and Post Treatment with Tradamixina.

**Fig. 4:**
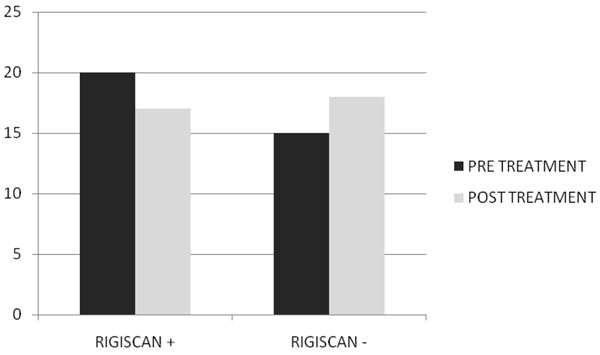
Rigiscan Results Pre and Post Treatment with Tadalafil 5mg Daily.

**Fig. 5:**
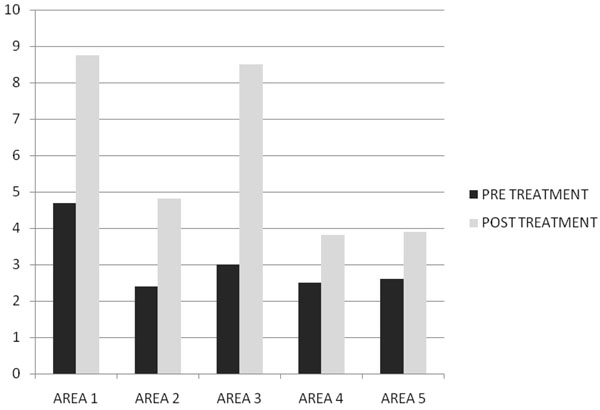
IIEF Score Pre and Post Treatment with Tradamixina.

**Fig. 6:**
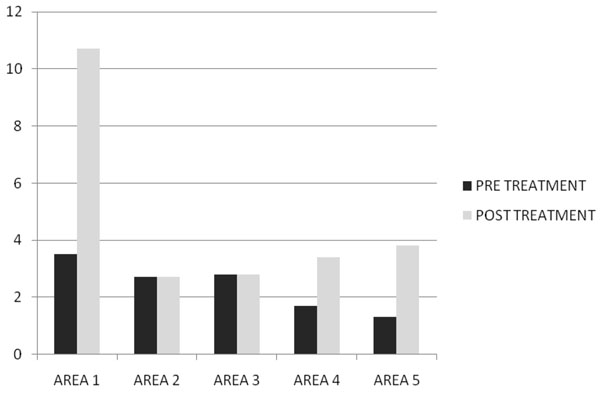
IIEF Score Pre and Post Treatment with Tadalafil 5mg Daily.

**Fig. 7:**
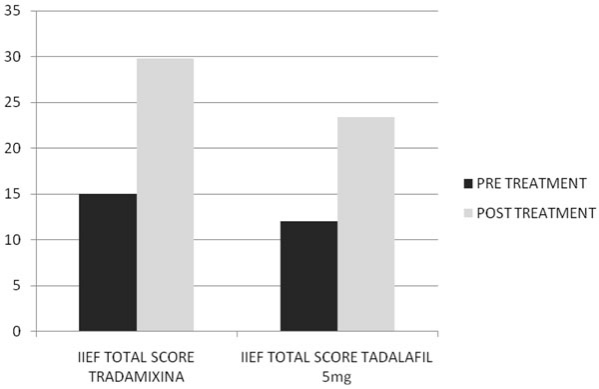
IIEF Total Score Ttradamixina vs Tadalafil mg 5 mg Daily.

**Fig. 8:**
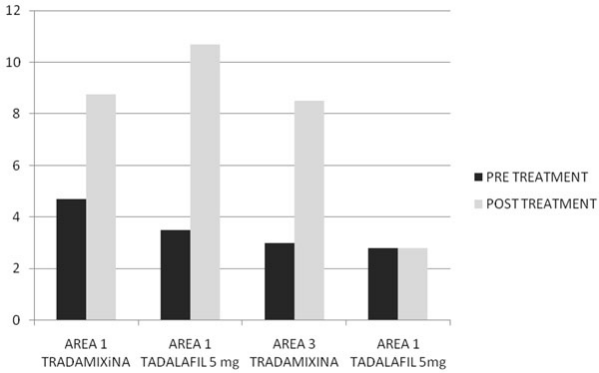
Area 1 and Area 3 Scores Pre and Post Treatment Ttradamixina vs Tadalafil 5mg Daily.

After two months of treatment in group A serum total testosterone levels (230±18 ng/dl vs 671±14 ng/dl ) and free testosterone levels(56± 2.4 pg/ml vs 120± 3.9pg/ml) with(p<0.005) increased, while in group B serum total testosterone levels (245±12 ng/dl vs 247±15 ng/dl ) and free testosterone levels(53± 0.3 pg/ml vs 55± 0.5pg/ml) with(p<0.005) increased but not statistically significant. In Fig. 1 and 2 we compared the increase of serum total testosterone levels and free testosterone levels in both groups. The group treated with tradamixina has increased total testosterone as well as free testosterone compared to the group treated with tadalafil. At visit patients were evaluated by instrumental examination (NPTR- nocturnal penile tumescence and rigidity test- with Rigiscan). At Pre-treatment there were 20 and 22 patients with positive Rigiscan in group A and group B respectively, while there were 15 and 13 patients with negative Rigiscan respectively. After the treatment in group A there were 17 patients with positive Rigiscan and 18 patients with negative Rigiscan, while in group B there were 10 patients with positive Rigiscan and 25 patients with negative Rigiscan (Fig 3-4). We asked all patients to complete the International Index of Erectile Function (IIEF) questionnaire. Fig 5 and 6 show the results of IIEF (singles areas); in group A and B respectively pre and post treatment. In group A (Fig 5) the singles areas scores increased after treatment (*area 1* 4.70±1.2 vs 8.75 ± 1.6; *area 2* 2.40 ±0.8 vs 4.80±1.0; *area 3* 3.0±0.9 vs 8.50±0.8; *area 4* 2.50±0.4 vs 3.80 ±0.9 ; *area 5* 2.60±0.4 vs 3.90±0.7.) with(p<0.005) , in group B (Fig 6) also , but with a different trend (*area 1* 3.50±1.2 vs 10.70 ± 1.5; *area 2* 2.70 ±0.7 vs 2.70±0.7; *area 3* 2.8±0.8 vs 2.80±0.8; *area 4* 2.70±0.2 vs 3.40 ±0.8 ; *area 5* 1.30±0.3 vs 3.80±0.60). The IIEF total score in group A increased after 60 days of treatment with tradamixina (15±1.5 vs 29.77±1.2); the total score in group B increased slightly(12±1.3 vs 23.40±1.2) (Fig 7), because in particular areas the score was not increased, particularly area 1( *group A* 4.70±1.2 vs 8.75± 1.6 and *group B* 3.50±1.2 vs 10.70 ± 1.5 ) and area 3 ( *group A* 3.0±0.9 vs 8.50±0.8 and *group B* 2.8±0.8 vs 2.80±0.8).The area 1 is erectile function while the area 3 the libido (Fig 8). We asked all patients to complete the Sexual quality of life Questionnarie-Male (SQoLM). The patients were asked to rate each statement according to how much they agree or disagree with it by circling one of the six categories. In group A the total score improved after treatment (16±2,3 vs 33±4,1(p<0.005). The total score in group B also improved (16±3,4 vs 31±2,1). In particular in category 2 *“When I think about my sexual life*, *I feel Depressed”* the improvement in group A was greater than in group B; infact in group A (2,5±0,3 vs 5,6±0,7(p<0.005) ) while in group B (2,3±0,6 vs 3,6±0,4(p<0.005) ). In category 5 “*When I think about my sexual life*, *I feel “anxious”* the improvement in group B was greater than in group A, infact in group A (2,3±0,6 vs 4,7±0,2(p<0.005) ) while in group B (1,5±0,6 vs 5,7±0,3(p<0.005) ). In category 6 : *“When I think about my sexual life*, *I feel like I have lost something”* there was an improvement statistically significant in both groups (group A 2,7±0,5 vs 5,9±0,1 ; group B 2,4±0,2 vs 5,7±0,3).( Tab.1). Tradamixina has no side effects, while the most common side effects with tadalafil were headache (n=5), followed by nasopharyngitis (n=4), back pain (n=4), dizziness (n=1), dyspepsia(n=2) (Tab.2)**.** The serum testosterone level improved in group A because the protodioscin has an androgen-mimetic action, binding and activating the receptor of testosterone. So this substance is able to increase the endogenous production of testosterone, dihydrotestosterone, a hormone luteinizing hormone (LH), dehydroepiandrosterone (DHEA) and dehydroepiandrosterone sulfate (DHEA-S) [30,31].

**Table 1:** Sexual Quality of Life Questionnarie-Male Tradamixina vs Tadalafil 5 mg Daily

Area	Tradamixina Pre treatment	Tradamixina Post treatment	Tadalafil 5 mg Pre treatment	Tadalafil 5 mg Post treatment
Area 1score	3,3±0,2	5,7±0,4	3,3±1,5	5,3±0,2

Area 2 score	2,5±0,3	5,6±0,7	2,4±0,6	5,6±0,4

Area 3 score	2,2±0,4	5.8±1,2	2,7±0,3	4,3±0,4

Area 4 score	3,0±0,3	5,3±1,5	3,7±0,2	5,2±0,3

Area 5 score	2,3±0,6	4,7±0,2	1,5±0,6	5,7±0,3

Area 6 score	2,7±0,5	5,9±0,1	2,4±0,2	5,7±0,3

Total score area	16±2,3	33±4,1	16±3,4	31±2,1

**Table 2:** Side Effects of Tradamixina vs Tadalafil 5 mg

Side effects	TRADAMIXINA (n=35)	TADALAFIL 5mg(n=35)
Headache	n=0	n=5

Nasopharyngitis	n=0	n=4

Back pain	n=0	n=4

dizziness	n=0	n=1

dyspepsia	n=0	n=2

Hormonal effects of Tribulus Terrestris (TT) were evaluated in primates, (rabbit and rat) to identify its usefulness in the management of erectile dysfunction (ED). TT extract was administered intravenously, as a bolus dose of 7.5, 15 and 30 mg/kg, in primates for acute study. Rabbits and normal rats were treated with 2.5, 5 and 10mg/kg of TT extract orally for 8 weeks, for chronic study. In addition, castrated rats were treated either with testosterone cypionate (10mg/kg, subcutaneously; biweekly for 8 weeks) or TT orally (5mg/kg daily for 8 weeks). Blood samples were analyzed for testosterone (T), dihydrotestosterone (DHT) and dehydroepiandrosterone sulphate (DHEAS) levels using radioimmunoassay. In primates, the increases in T (52%), DHT (31%) and DHEAS (29%) at 7.5mg/kg were statistically significant. In rabbits, both T and DHT were increased compared to control, however, only the increases in DHT (by 30% and 32% at 5 and 10mg/kg) were statistically significant. In castrated rats, increases in T levels by 51% and 25% were observed with T and TT extract respectively that were statistically significant. TT increases some of the sex hormones, possibly due to the presence of protodioscin in the extract. TT may be useful in mild to moderate cases of ED [32]. Tadalafil instead of tradamixina doesn’t increased serum testosterone levels. In literature there isn’t proof of improvement. However sustained improvement in sexual function after 12 months of tadalafil administration is associated with increased T:E ratio mainly related to reduction of E levels. We hypothesize that androgen-estrogen cross-talk and possible inhibition of aromatase activity during chronic exposure to tadalafil might have a role in the regulation of erectile function [43]. The IIEF total score improved in group A, because in group B there is a different trend in a particular area, like area 1 and area 3. In fact in area 1: erectile function tadalafil shows greater efficacy in men previously naïve to PDE5−inhibitor therapy. Significantly higher proportions of subjects receiving once-daily tadalafil (vs. placebo) reported improved erections (GAQ1- Global Assessment Questions and sex lives) (GAQ2- Global Assessment Questions). Once-daily tadalafil treatment also resulted in: 1) significantly higher treatment satisfaction on the EDITS (the Erectile Dysfunction Inventory of Satisfaction) at endpoint; and 2) significantly improved psychosocial outcomes, as indicated by increases in the total score of the SEAR (the Self-Esteem and Relationship questionnaire) and three of the four SEAR subdomains compared to placebo. Previous positive findings for psychosocial outcomes (e.g. sexual self-confidence) and treatment satisfaction on the EDITS have also been reported in 20 studies of on-demand tadalafil [44]. Tradamixina, however, shows improvement in the same areas because Biovis contains polymers of d-glucosamine and n-acetyl-d-glucosamine that act on both the non-adrenergic and non-colinergic system (NANC) and on the endothelial cell system as a strong nitric oxide synthetase (NOS) stimulator [32]. Evidence is accumulating that is the CNS, NO function is essential for erectile responses (Fig. 2). NO can modulate sexual behavior and penile erection [49] and may act in several discrete brain regions, eg in the MPOA63, 64 and the PVN. The area 3 – libido improved in group A. In fact Tribulus Terrestris is able to increase the endogenous production of testosterone, dihydrotestosterone, a hormone luteinizing hormone (LH), dehydroepiandrosterone (DHEA) and dehydroepiandrosterone sulfate (DHEA-S) [30,31], and so the reduced libido is widely considered the most prominent symptomatic reflection of low testosterone (T) levels in men [1,2]. Ecklonia bicyclis has radical scavenger activity and has antioxidant properties [30], so it protects the elderly man against aging. Indeed Tribulus Terrestris, Ecklonia bicyclis and polymers of d-glucosamine and n-acetyl-d-glucosamine have a synergic anti-aging action [50]. Tadalafil improved the erectile function but doesn’t improve the libido. The IIEF total score is higher in group A. The result is guaranteed by Tribulus Terrestris. In elderly men, decreased libido and impotence present a common and important clinical problem, sexual dysfunction (encompassing erectile dysfunction, ejaculatory disorders and loss of libido) is highly prevalent in ageing men and can have substantial adverse effects on their quality of life [51-54]. In fact testosrerone replacement may have an antidepressant effect in depressed patients, especially those with hypogonadism [55]. Tadalafil improved Rigiscan examination and it inhibits PDE-5 at very low concentrations. The efficacy of PDE-5 inhibitors varies from 40% to 85% depending on the severity and etiology of ED [56]. As the mechanism of the PDE-5 inhibitor class requires sufficient NO release mediated through sexual stimulation, it is not surprising that there is a “learning" effect in some patients who are reinitiating sexual activity; although about two thirds of patients respond within the first two doses, the others only begin to respond on subsequent dosing reaching a maximum threshold of response for the study population after about 6–8 doses [57].

## Conclusion

The treatment twice a day with a new compound “Tradamixina” (150 mg of Alga Ecklonia Bicyclis, 396 mg of Tribulus Terrestris and 144 mg of D-Glucosamine and N-Acetyl-D-Glucosamine) for two months improved libido in elderly men, with particular symptom of LOH which can have substantial adverse effects on their quality of life. This compound is effective in the treatment of probable mild to moderate erectile dysfunction in LOH and provides a clear synergistic effect with the administration of the 3 substances in a single capsule. It can be used to improve in a natural way the male sexual function due to its antioxidant, antifibrotic and anti-aging action. Tadalafil improves erectile function but not libido, so in LOH it is not effective for the symptoms. Unlike Tadalafil, Tradamixina doesn’t cause side effects and the aim of a valid treatment for these patients is to “change” the artificial link “pill-sexuality”- ”pill on demand” reaching the natural sexual sequence desire-excitation-erection, through a direct action on the erectile disfunction pathogenesis. So Tradamixina can be used in LOH for its symptomatic and preventive action.

## List of abbreviations

CV: Coefficient of variation; DHEA: Dehydroepiandrosterone; DHEA-S: Dehydroepiandrosterone sulfate; ED: Erectile dysfunction; EDITS: Erectile Dysfunction Inventory of Satisfaction; GAQ1: Global Assessment Questions and sex lives; GAQ2: Global Assessment Questions; HDL: High Density Lipoprotein; IIEF-EF: International Index of Erectile Function−Erectile Function domain; LOH: Late-Onset Hypogonadism; NANC: Non-adrenergic and non-colinergic system; NO: Nitric oxide; NOS: Nitric oxide synthetase; NPTR: Nocturnal penile tumescence and rigidity test; NR: Negative Rigiscan; PDE5: Phosphodiesterase type 5; PR: Positive Rigiscan; RL: Reduced libido; SEAR: Self-Esteem and Relationship questionnaire; SEP: Sexual Encounter Profile; SHBG: Sex hormone-binding globulin; SQoLM: Sexual quality of life Male questionnaire; T: Testosterone; TD: Testosterone deficiency; TT: Tribulus terrestris.

## Competing interests

The authors declare that they have no competing interests

## Authors’ contributions

FI: conception and design, interpretation of data, given final approval of the version to be published; DP: conception and design, interpretation of data, given final approval of the version to be published; EI: acquisition of data, drafting the manuscript, given final approval of the version to be published; GR: acquisition of data, drafting the manuscript, given final approval of the version to be published; AR: acquisition of data, drafting the manuscript, given final approval of the version to be published; BA: critical revision, interpretation of data, given final approval of the version to be published.
